# Extreme Environment *Streptomyces*: Potential Sources for New Antibacterial and Anticancer Drug Leads?

**DOI:** 10.1155/2019/5283948

**Published:** 2019-07-01

**Authors:** Periyasamy Sivalingam, Kui Hong, John Pote, Kandasamy Prabakar

**Affiliations:** ^1^Department F.-A. Forel for Environmental and Aquatic Sciences and Institute of Environmental Sciences, School of Earth and Environmental Sciences, Faculty of Science, University of Geneva, Uni Carl Vogt, 66 Boulevard Carl-Vogt, CH-1211 Geneva 4, Switzerland; ^2^Key Laboratory of Combinatorial Biosynthesis and Drug Discovery (Wuhan University), Ministry of Education, Wuhan University School of Pharmaceutical Sciences, Wuhan 430071, China; ^3^Postgraduate and Research Department of Zoology, Jamal Mohamed College, Tiruchirappalli 620020, Tamil Nadu, India

## Abstract

Antimicrobial resistance (AR) is recognized as one of the greatest threats to public health and in global concern. Consequently, the increased morbidity and mortality, which are associated with multidrug resistance bacteria, urgently require the discovery of novel and more efficient drugs. Conversely, cancer is a growing complex human disease that demands new drugs with no or fewer side effects. Most of the drugs currently used in the health care systems were of *Streptomyces* origin or their synthetic forms. Natural product researches from *Streptomyces* have been genuinely spectacular over the recent years from extreme environments. It is because of technical advances in isolation, fermentation, spectroscopy, and genomic studies which led to the efficient recovering of *Streptomyces* and their new chemical compounds with distinct activities. Expanding the use of the last line of antibiotics and demand for new drugs will continue to play an essential role for the potent *Streptomyces* from previously unexplored environmental sources. In this context, deep-sea, desert, cryo, and volcanic environments have proven to be a unique habitat of more extreme, and of their adaptation to extreme living, environments attribute to novel antibiotics. Extreme *Streptomyces* have been an excellent source of a new class of compounds which include alkaloids, angucycline, macrolide, and peptides. This review covers novel drug leads with antibacterial and cytotoxic activities isolated from deep-sea, desert, cryo, and volcanic environment *Streptomyces* from 2009 to 2019. The structure and chemical classes of the compounds, their relevant bioactivities, and the sources of organisms are presented.

## 1. Introduction

*Streptomyces* are Gram-positive and have high G + C DNA content with a complex life cycle having the potential to produce many clinically important bioactive molecules. Among Gram-positive bacteria, *Streptomyces* represents a significant source for supplying bioactive natural products with clinical and pharmaceutical applications. Notably, *Streptomyces* accounts for 39% of all microbial metabolites, and in Streptomycetales class, this genus alone reported to produce nearly 80% of bioactive molecules [1]. For the genus *Streptomyces*, there are more than 800 species with validly published names (http://www.bacterio.net/Streptomyces.html [2]). Historically, *Streptomyces* from environmental sources has been pivotal in the discovery of important bioactive secondary metabolites including antibiotics, immunosuppressive drugs, anticancer drugs, and other biologically active compounds [3–6]. However, depressingly in the last decades, the continual rediscovery of similar and known compounds from terrestrial *Streptomyces* has resulted. Therefore, it is advantageous to the search for potential microorganisms from unexplored or underexploited natural environments as a source of new bioactive molecules [7–9]. Considering this, in recent years, much of the attention focused on more extreme environment habitats such as deep-sea, desert, cryo, and volcanic environments for the isolation of potential *Streptomyces* species. Until recently, most of the compounds from genus *Streptomyces* have been isolated by culture-dependent methods rather than by the metagenomic approach. The culture-dependent approach has been demonstrated to have convincing reasons to study the species behaviour and to use many strategically correct procedures such as one strain many compounds (OSMAC) [10] to isolate novel compounds. Thus, it is the hope that cultivation-based approaches would expand our knowledge in an unprecedented way for the new drug development, genome study, and combinatorial biosynthesis. As evidenced above, *Streptomyces* is an undoubtedly potent genus to hunt for novel pharmaceutically essential compounds derived from underexplored extreme environment habitats for next-generation drugs to counteract the worldwide increase of drug resistance and to meet the demand for novel drugs with no or fewer side effects.

## 2. Deep Sea

Marine ecosystem so far is the most significant known environment on this planet [11]. Of the total marine ecosystem, more than 90% is designated as deep sea characterised with many distinct features [12] that attributed for individual species distribution [13] and an important resource for bioactive molecule discovery. The ocean covers 70% of the total world's surface, and the majority of it is below 1000 meters of depth [14]. It has been documented that the world's ocean contained 16 trenches which are having a depth deeper than 7000 m (submarine_topographical_features#List_of_oceanic_trenches).

Deep-sea oceans are the most extreme environments on Earth. Skropeta [14] reported that deep sea is the place with the highest richness in biodiversity, surpassing the rain forests and the coral reef. Organisms inhabiting in the deep sea can cope with such harsh conditions in the absence of light and under low percentage of oxygen and extremely high pressures, requiring several adaptations in terms of biochemical and physiological processes [14]. These special environment variables may lead to producing distinct chemical entities with diverse biological activities. The first article which emphasises on the isolation of natural compound from deep-sea-derived *Streptomyces* (DSDS) was published in 1995 [15]. After that, this environment was abandoned for nearly a decade. However, since late 2005, the exploration of deep-sea *Streptomyces* has been steadily growing on. Though the number of publications in connection with deep-sea *Streptomyces*-derived natural compounds has not been in more significant numbers, emphasis on structural diversity and biological activity made it a crucial extreme habitat to pursue this resource for novel compounds to meet the need of the 21st century. Indeed, it is beyond our expectations even more that actinomycetes have been isolated from Mariana trench, at 10,898 m [7].

## 3. Desert

Remarkably, one-fifth of our planet Earth is covered by desert which has been emphasised by devoid of vegetation or low and extremely low and unpredictable rainfall [16]. Desert is further characterised by arid conditions including high UV radiation, extreme temperatures and desiccation, high salinity, the presence of inorganic oxidants, deficient concentrations of organic carbon, and physical instability caused by strong winds [17–19]. It has been proposed that the Atacama is the oldest and driest known desert among others on the Earth and as an accurate analogue of Martian soils [20]. Unlike deep-sea environments, desert habitat has gained tremendous importance in the last decade for the search of such prolific *Streptomyces* sp. in the context of natural product discovery [21, 22]. Given the unusual climatic conditions, the desert have been believed to home for unique potential *Streptomyces* which are mostly yet to be explored to neutralise the emerging drug-resistant infectious diseases and cancer with their novel bioactive molecules. While considering the recent and past studies [23, 24], it becomes clear that the Atacama Desert is focused consistently than other deserts and many more reports to come from other regions.

## 4. Extremely Low Cold or Cryoenvironment

Extremely low cold or cryoenvironment is an inexhaustible microbial habitat which has been emphasised by several studies in recent years [25, 26]. Because of significant climatic variables in extreme cold habitats, microbes inhabiting there can adapt to harsh conditions which can, in turn, produce novel compounds that are valuable for biotechnology applications [27]. Extreme low cold temperature prevails on Earth in Polar Regions of Arctic and Antarctic, Siberia, Himalayan Mountains, and some permafrost. Bhave et al. [28] reported that Antarctica is the coldest, driest, and windiest continent on Earth. Besides, high UV exposure and low organic and high salt concentrations in soils of Antarctica render them an unusual environment [28]. Arctic is another polar region which has been geographically isolated for millions of years on Earth [29] and has been emphasised by cold winter and cool summer [30], the presence of low nutrient concentrations, high UV radiation, and extreme capricious in day length [27]. The Himalayan cold deserts are reported to have a fragile ecosystem and complex climate [31]. The possible occurrence of high-intensity UV radiation in the lofty mountain is also evidenced in the past study [32]. Therefore, to isolate biotechnologically important *Streptomyces* spp. from the most poorly explored cryoenvironments warrants for new molecules with potential applications. As these environments considered being the greatest diversity of culturable actinomycetes, studies in the recent past revealed the occurrence of novel *Streptomyces* spp. from the Antarctic ecosystem [33, 34].

## 5. Volcanic Environment

It is evident that volcanic spring is one of the extreme habitats on Earth and harbours novel microbes as a source of potential drug leads. Volcanic habitats have been neglected over the years and just a few years ago have attracted considerable interest among the researchers. In evidence, it has been documented earlier that volcanic islands have potential harbour microorganisms with distinct genetic features for secondary metabolite production [35]. To date, however, volcanic environments are the least explored and remained one of the understudied extreme environments among the others which provide a tremendous avenue for the search of new bioactive molecules derived from *Streptomyces* species. Perhaps, until recently, only a very few studies have been performed concerning the isolation of bioactive natural products derived from volcanic environment *Streptomyces* [35–37]. Notably, these studies have been undertaken within the last six years.

## 6. Recent Advancements in Cultivation and Classification of *Streptomyces* from Extreme Environments

Given the bottleneck that exists with real lab isolation strategies of extreme habitat *Streptomyces*, the new technologies are continually evolving, and actinomycetes researchers have already been made on that front to explore extreme habitats for natural drug discovery by advanced culture-dependent methods. However, it has been proposed that extreme environment microbes do not require extreme culture conditions such as extreme temperature, pH, and pressure [38]. Firstly, to access the novel *Streptomyces* from extreme habitats will be the key to identify and characterise the molecules with the potential application [10]. In this context, a polyphasic taxonomic approach which includes the biochemical, phenotypic traits and molecular methods in an integrative manner for detecting new *Streptomyces* sp. from unexploited environments and dereplication may prove worthwhile. These methods may prevent the reinvestigation of previously reported strains [10].

Until recently, the discovery of bioactive compounds from *Streptomyces* has been confined to a process of bioassay-guided identification of bioactive fraction from fermented cultures under a defined set of culture conditions. The advanced comprehensive spectroscopy including LC-MS and NMR [39, 40], genome mining approach [41], whole-genome sequencing (WGS), next-generation sequencing (NGS), and bioinformatics tools such as AntiSMASH, BAGEL, SBSPKS and SMURF, and MIBiG led to the isolation of compounds and biosynthetic gene clusters (BGC) from potential strains [42–44]. The identified cryptic/silent gene cluster can be activated for their likely compounds production by changing culture parameters due to their missing environmental cues [42]. Nowadays, reductions in cost and advances in DNA sequencing technology have removed many of the barriers to acquiring the genome sequence of *Streptomyces*. It has been demonstrated that the available genome sequences of most actinomycetes contain more than 20 BGCs [41]. Therefore, the identification of biosynthetic genes from *Streptomyces* which tend to be colocalized in the chromosome as biosynthetic gene clusters is a promising target to study molecular biology, metabolic engineering, and heterologous expression of new compounds.

## 7. Deep-Sea *Streptomyces* Isolation

Earlier dedicated sampling and culture-dependent studies strongly suggest that *Streptomyces* species are dwelling in considerable number in deep-sea sediment samples [7, 41, 45–48]. However, until now a very few natural compounds have been isolated from deep-sea-derived *Streptomyces*. It is because of the limitation in sample collection technology and following isolation methods in real laboratory settings. To effectively collect the samples from deep sea, various advanced collection devices have been made and they have been well documented [49]. The primary devices among these are the remote-operated submarine vehicle (ROVs) [7] and autonomous underwater vehicles (AUVs) [50] that strikingly breakthrough the impediment to access the deep-sea samples. Next, to the sample collection, transportation to the laboratory and therein storage of sediment samples at −80°C for a more extended period find better in the recovering of *Streptomyces* by preventing fast-growing bacteria [51]. In the preliminary isolation steps, pretreatment and serial dilutions of sediment samples have been shown to be useful in the enumeration of spore-forming actinobacteria [7, 52]. It can also be crucial to use ideal media and seawater in the isolation media [53] and antibiotics such as nystatin and rifampicin to inhibit the fungal and nonfilamentous bacterial growth [54]. Incubation temperature and time have also been known to influence the isolation of deep-sea *Streptomyces* [52–54]. These innovative and improved technologies paved the way for the exploration of *Streptomyces* from deep-sea habitats and eventually substantiated by various dedicated studies which involve active culture-dependent microbiological experimentation [41, 46–48]. It has also been reported that *Streptomyces* is the most dominant species in marine sediments with an increase of depth [53]. Notably, to date, many potential natural compounds with unique structures from *Streptomyces* inhabiting the South China Sea have been isolated than any other deep-sea environments.

## 8. Isolation of *Streptomyces* from Desert

Given published articles so far, it can be explained that the Atacama Desert has gained more interest than other deserts. Studies have demonstrated that extreme habitat of hyperarid or an absolute desert has revealed the presence of culturable and novel *Streptomyces* [23, 55]. Okoro et al. reported that the cultivable percentage of genus *Streptomyces* is about 91% from the soil sample collected in the Atacama Desert among other actinomycetes [17]. Nonetheless, there was limited number of studies conducted with regard to the isolation of *Streptomyces* spp. from the Thar Desert, India, for their bioactive potential [56–58], and notably, no purified compounds with their chemical structures have been reported yet. Recently, Tiwari et al. reported the extracts of *Streptomyces* spp. isolated from the Thar Desert, displaying a promising inhibitory activity against multidrug-resistant*Streptococcus pneumoniae* [58]. Selective isolation procedures including serial dilution followed by dry heat at 55°C for 6 minutes for soil samples collected from desert environments are proved to be useful about the isolation of actinomycetes and diversity [17]. It has also shown that pretreatment of the soil sample subject to air drying at 50°C and preincubation at 50°C for an hour yielded *Streptomyces* on ISP2 media [59]. Hozzien et al. reported that minimal media (MM) containing glucose, yeast extract, and mineral salts which might be useful for selective isolation of actinomycetes including *Streptomyces* from the desert soil with other media were used [60]. Raffinose-histidine agar supplemented with antibiotics such as cycloheximide (25 *µ*g·ml^−1^) and nystatin (25 *µ*g·ml^−1^) was also found to apply for the isolation of novel species of *Streptomyces* [61]. Selective media such as Gauze's No. 1 medium [62], humic acid-vitamin agar, SM1 agar, and starch casein agar [22, 63] have been used to isolate new *Streptomyces* sp. which can be used to derive new compounds.

*Streptomyces violaceusniger* strain SPC6 isolated from the Linze Desert has been found to grow in media supplemented with 0 M to 1 M·NaCl, which indicates its adaptation to the arid desert environment [64]. Remarkably, this strain had also shown a high growth rate and short life cycle with just two days at 37°C. It was noted that the optimal growth temperature is ranging from 28°C to 30°C suitable in the context of isolation of *Streptomyces* species from desert soils [59, 65]. The incubation time has been reported ranging from two weeks to four weeks [65–67].

## 9. Isolation of *Streptomyces* from Cryoenvironments

Few past studies have demonstrated that the existence of novel *Streptomyces* spp. from the Antarctic ecosystem and other distinct studies requires to be investigated in such environmental sources [33, 34]. Likewise, recently published papers describe novel *Streptomyces* isolated from Arctic glacier [25, 26]. But, till date, no studies described yet concerning novel *Streptomyces* spp. isolated from Himalayan harsh environments. However, a minimal investigation of this habitat has been undertaken. Several factors are considered for the isolation of *Streptomyces* from cryoenvironment samples. They include immediate storage at below 0°C [33], transportation at below 0°C [33], selective isolation media such as tryptone-yeast extract (TY) agar actinomycete isolation medium (1 L of seawater, 18 g of agar, 20 mg/L of cycloheximide, 20 mg/L of nystatin, and 10 mg/L of nalidixic acid), and starch-casein-nitrate agar [33, 68, 69], incubation temperature between 18°C and 28°C, and incubation time ranging from one week to a month [29, 34, 69].

## 10. Isolation of *Streptomyces* from Volcanic Environment

Studies have demonstrated that the presence of indigenous and distinct species of *Streptomyces* that drive uniqueness to the volcanic habitat is indicative for future exploration. Importantly, although there have been few notable studies on isolation of natural drugs from volcanic *Streptomyces* reported by Um et al., 2013, Cha et al., 2015, and Son et al., 2018 [35–37], the knowledge of *Streptomyces* population in volcanic habitat is sparse. It has been postulated that serial dilution of samples, humic acid-vitamin agar (HV) supplemented with nystatin (50 mg·l^−1^) and nalidixic acid (20 mg·l^−1^), and prolonged incubation time over three weeks are proven to be useful in the isolation of *Streptomyces* sp. from volcanic habitats [70].

## 11. Novel Antibacterial and Anticancer Compounds from Cultured Deep-Sea *Streptomyces*

**Figure 1:**
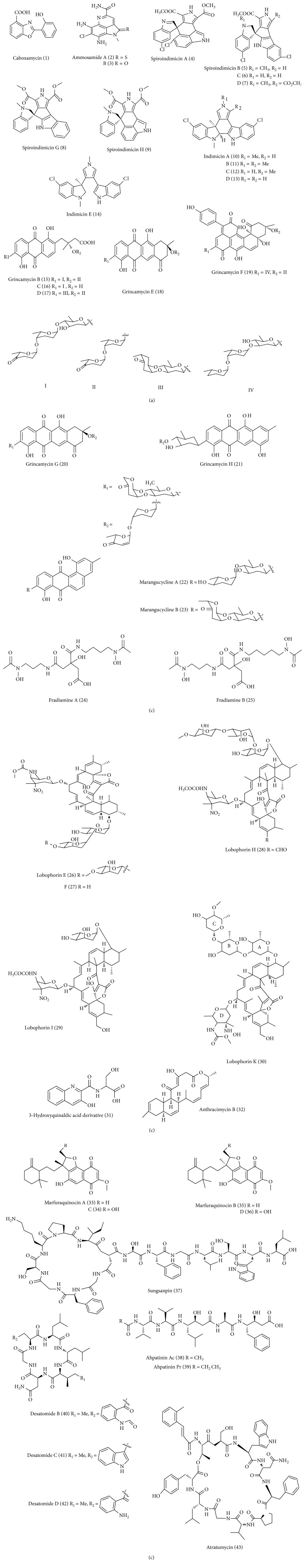
Deep-sea *Streptomyces*-derived novel antibacterial and anticancer compounds.

Table 1 presents the novel compounds derived from deep-sea *Streptomyces* (DSDS), and their corresponding structures are shown in Figure 1.

### 11.1. Benzoxazole

A new antibiotic named caboxamycin (1) belonging to the benzoxazole class is produced by *Streptomyces* sp. NTK 937, isolated from deep sediments collected at a depth of 3814 m near Canary Islands [71]. Caboxamycin displayed antibacterial activity against Gram-positive bacteria, antitumor activity against AGS, MCF7, and HepG2, and enzyme inhibitory activity against phosphodiesterase.

### 11.2. Pyrroloiminoquinone

Ammosamides A (2) and B (3) are belonging to the pyrroloiminoquinone class produced by *Streptomyces* sp. CNR-698. The strain was isolated from the deep-sea sediment collected at a depth of 1618 m in Bahamas Islands. Ammosamides A and B displayed in vitro cytotoxicity activity against colon carcinoma cell line HCT-116, with the IC_50_ value of 320 nM [72].

### 11.3. Alkaloids

*Streptomyces* sp. SCSIO 03032, isolated from a deep-sea sediment sample collected at a depth of 3412 m in the South China Sea, yielded four new bisindole alkaloids spiroindimicins A–D (4–7). Spiroindimicin B showed moderate cytotoxic activities against several cancer cell lines including CCRF-CEM, B16, and H460 with IC_50_ values of 4, 5, and 12 *μ*g/mL, respectively. Spiroindimicin C had shown inhibitory activity against HepG2 and H460 with IC_50_ values of 6 and 15 *μ*g/mL, respectively. Spiroindimicin D displayed moderate inhibitory activity against HepG2, B16, and H460 [73]. The presence of the (5, 5) spiro ring system in spiroindimicins B–D might have contributed moderate antitumor activities [73].

The inactivation of halogenase gene spmH in *Streptomyces* sp. SCSIO 03032 yielded two new bisindole alkaloids named spiroindimicins G (8) and H (9) [84]. Spiroindimicin G showed moderate cytotoxic activities against four cancer cell lines including SF-268, MCF-7, HepG2, and A549 with IC_50_ values of 16.09 ± 1.26, 19.11 ± 2.23, 13.57 ± 0.24, and 10.28 ± 0.14 *μ*M, respectively. Spiroindimicin H also displayed moderate inhibitory activity against SF-268, MCF-7, HepG2, and A549 with IC_50_ values of 23.54 ± 0.29, 33.02 ± 3.41, 20.92 ± 0.69, and 18.16 ± 0.59, respectively.

Indimicins A–E (10–14) are new bisindole alkaloids antibiotics bearing a unique 1′,3′-dimethyl-2′-hydroindole moiety along with two new compounds lynamicins F and G obtained from the fermentation broth of deep-sea *Streptomyces* sp. SCSIO 03032, isolated from the Bay of Bengal and Indian Ocean, at a depth of 3412 m [74]. Among five, indimicin B alone had shown in vitro cytotoxic activity against MCF-7 with an IC_50_ greater than 10.0 *µ*M. But indimicin B did not exhibit cytotoxicity against NCI–H460, HepG2, and SF268 [74].

### 11.4. Angucycline

Grincamycins B–F (15–19) are belonging to new glycoside angucycline antibiotics obtained from the culture broth of *Streptomyces lusitanus* SCSIO LR32, isolated from the South China Sea at a depth of 3370 m. All but except grincamycin F showed in vitro cytotoxicity activity against human cancer cell lines such as HepG2, SW-1990, and MCF-7 and the mouse melanoma cell line B16, with the IC_50_ values ranging from 1.1 to 31 *µ*M [75]. It has shown that grincamycin F differs from grincamycin primarily in the structure of its enlarged aglycone, which contains a six-membered lactone ring and a hydroxybenzene in addition to the typical angucycline four-ring system. The investigators revealed that the enlarged aglycone of grincamycin might eliminate its cytotoxicity properties [75].

*Streptomyces lusitanus* SCSIO LR32, isolated from the South China Sea at a depth of 3370 m, yielded two new compounds named grincamycins G (20) and H (21) belonging to rearranged linear angucycline glycosides. Intriguingly, the new compound grincamycin H showed cytotoxicity on Jurkat T cells with an IC_50_ value of 3.0 *µ*m. However, grincamycin G exhibited no cytotoxic activity at the concentration of 20 *µ*m on Jurkat T cells [81]. The authors ascertain that aglycone moiety may also have a role in the derivation of chemical and biological diversity of angucycline in addition to the sugar unit.

*Streptomyces* sp. SCSIO 11594, isolated from a deep-sea sediment sample collected at a depth of 2403 m in the South China Sea, yielded two new C-glycoside angucycline antibiotics, namely, marangucyclines A (22) and B (23) together with three known compounds dehydroxyaquayamycin, undecylprodigiosin, and metacycloprodigiosin [47]. All the compounds were tested for cytotoxicity activity against four cancer cell lines A594, CNE2, HepG2, and MCF-7. Marangucycline B and undecylprodigiosin displayed promising cytotoxic activity against all cancer lines. The investigators reported that marangucycline B presented 20-fold more cytotoxic activity than cisplatin, while undecylprodigiosin showed tenfold more cytotoxicity than cisplatin which is used as positive control. The keto sugar of marangucycline B is believed to be a possible reason for significant cytotoxicity activity with IC_50_ values ranging from 0.24 to 0.56 *μ*M. Marangucycline B showed an IC_50_ value of 3.67 *μ*M against noncancerous hepatic cell line HL7702, which indicates its cancer cell selectivity.

The investigators further reported that marangucyclines A and B and dehydroxyaquayamycin exerted weak antibacterial activities against *Enterococcus faecalis* ATCC 29212 with a MIC value of 64.0 *μ*g/mL. Dehydroxyaquayamycin showed selective inhibitory activity against methicillin-resistant *Staphylococcus epidermidis*shhs-E1 with a MIC value of 16.0 *μ*g/mL [47].

### 11.5. Siderophore

A new siderophore and its derivative, designated as fradiamines A (24) and B (25), were recently found to be produced by *Streptomyces fradiae* MM456M-mF7, isolated from the deep-sea sediment sample collected at a depth of 806 m in the Sagami Bay, Japan. Fradiamines A and B displayed moderate antibacterial activity against *Clostridium difficile* BAA-1382 with IC_50_ values of 32 and 8 *μ*g·ml^−1^, respectively [82].

### 11.6. Spirotetronate

A sea-derived *Streptomyces* sp. SCSIO 01127 recovered from a sediment sample collected at a depth of 1350 m in the South China Sea yielded two new antibiotics belonging to spirotetronate named lobophorins E (26) and F (27) together with two known analogues of lobophorins A and B [76]. Lobophorin F displayed potent antibacterial activities against Gram-positive *Staphylococcus aureus* ATCC 29213 and *Enterococcus faecalis* ATCC 29212. Lobophorin F also exhibited cytotoxic activities against SF-268, MCF-7, and NCI–H460 with IC_50_ of 6.82, 2.93, and 3.16 *µ*M, respectively [76]. It has been predicted that the absence of C-32 hydroxyl group in lobophorins E and F when compared with lobophorin B significantly enhances their antimicrobial properties against *S. aureus* ATCC 29213. Lobophorin E displayed antibacterial activity against *Staphylococcus aureus* ATCC 29213 with the MIC value of 32 *µ*g/mL. It has also been inferred from the structures that the presence of the terminal sugar moiety (4-*O*-L-digitoxose, sugar C) is disadvantageous for the antimicrobial and antitumor property. The investigators suggest that the presence of the nitro-sugar moiety is critical and change of sugar moieties will yield natural products with defined or altered biological activity [76].

*Streptomyces* sp. 12A35, recovered from a deep-sea sediment sample of South China Sea at a depth 2134 m, yielded two new spirotetronate antibiotics, namely, lobophorins H (28) and I (29) together with three known analogues, *O*-*β*-kijanosyl-(1 ⟶ 17)-kijanolide and lobophorins B and F [46]. Lobophorins H and I did not exhibit inhibitory activity against Gram-negative bacteria (*E. coli*) and fungi (*C. albicans* and *F. moniliforme*), whereas lobophorin H and lobophorin F showed moderate inhibitory activities against *Staphylococcus aureus* ATCC 29213 with the MIC values of 50 and 6.25 *μ*g/mL, respectively. Intriguingly, all the tested compounds displayed inhibitory activities against *Bacillus subtilis* CMCC 63501. Lobophorin H and lobophorin B displayed strong inhibitory activities against *Bacillus subtilis* CMCC 63501 with MIC values of 3.13 and 1.57 *μ*g/mL, respectively. On the contrary, lobophorin I, kijanolide, and lobophorin F showed moderate inhibitory activities against *Bacillus subtilis* CMCC 63501 with MIC values of 6.25, 50, and 50 *μ*g/mL, respectively. From the results, it was proposed that the monosaccharide units might play an essential role in the antimicrobial activity of lobophorins. The investigators also suggested that the increasing amount of monosaccharide units resulted in increased inhibitory activity. Thus, the potent antibacterial activity efficiency of lobophorin I and H against Gram-positive bacteria may provide the new candidature for anti-infective drug development [46].

*Streptomyces* sp. M-207, isolated from the deep-sea coral *Lophelia pertusa* collected at 1800 m depth in the central Cantabrian Sea, was found to produce a novel compound belonging to lobophorin family, designated as lobophorin K (30). Remarkably, lobophorin K exhibited cytotoxic activity on a human breast adenocarcinoma cell line (MCF-7), a human pancreatic carcinoma cell line (MiaPaca-2), and a human immortalised hepatocyte cell line (THLE-2) with IC_50_ values of 23.0 ± 8.9, 34.0 ± 85.1, and 6.3 ± 8.2 *µ*M, respectively [83]. Lobophorin K had also displayed a moderate and selective antibacterial activity against pathogenic methicillin-sensitive *Staphylococcus aureus* EPI1167 MSSA.

### 11.7. Hydroxyquinaldic Acid

*Streptomyces cyaneofuscatus* M-157, isolated from the deep sea at 1800 m depth in the central Cantabrian Sea, was found to produce a novel antibiotic 3-hydroxyquinaldic acid derivative (31). The compound exhibited cytotoxic activity on HepG2 with an IC_50_ value of 51.5 *μ*M [85].

### 11.8. Macrolide

*Streptomyces cyaneofuscatus* M-169, isolated from the deep-sea coral Gorgonacea collected at 1500 m depth in the central Cantabrian Sea, was found to produce a novel compound belonging to macrolide family, designated as anthracimycin B (32) [48]. Anthracimycin B displayed antimicrobial activity against *S. aureus* MRSA (methicillin-resistant) (0.33–0.65 *μ*M), *S. aureus* MSSA (methicillin-resistant) (10.5–20.9 *μ*M), vancomycin-sensitive*Enterococcu*s *faecium* (VANS) (0.33–0.65 *μ*M), vancomycin-sensitive *Enterococcus faecalis* (0.65-1.26 *μ*M), *Escherichia coli* (>41.8 *μ*M), and *Klebsiella pneumoniae* (>41.8 *μ*M) [48]. The authors also proposed that the presence of the methyl group at C-2 in anthracimycin B could be responsible for its potent antimicrobial activity.

## 12. Terpene

### 12.1. Sesquiterpenoid Naphthoquinones

A deep-sea-derived *Streptomyces niveus* SCSIO 3406 recovered from a sediment sample collected at a depth of 3536 m in the South China Sea yielded four new antibiotics belonging to sesquiterpenoid naphthoquinones named marfuraquinocins A−D (33-36) together with two other new geranylated phenazines named phenaziterpenes A and B. Marfuraquinocins A and C displayed cytotoxicity activity against NCI–H460 cancer cell line with IC_50_ values of 3.7 and 4.4 *μ*M, respectively. Marfuraquinocins A, C, and D showed antibacterial activities against *Staphylococcus aureus* ATCC 29213 with equivalent MIC values of 8.0 *μ*g/mL. Intriguingly, marfuraquinocins C and D showed antibacterial activity against methicillin-resistant*Staphylococcus epidermidis* (MRSE) shhs-E1 with MIC values of 8.0 *μ*g/mL [77].

### 12.2. Peptide

Sungsanpin (37) is a new lasso peptide (15 amino-acid) obtained from *Streptomyces* sp. SNJ013. The producing strain was recovered from a sediment sample collected at a depth of 138 m off the coast of Sungsanpo on Jeju Island, Republic of Korea. Sungsanpin showed inhibitory activity in a cell invasion assay for the lung cancer cell line A549 [78]. Sungsanpin is currently in preclinical trials for cancer treatment [50].

Two new linear peptides named ahpatinin Ac (38) and ahpatinin Pr (39) obtained together with the known ahpatinin iBu, pepstatin Ac, pepstatin Pr, and pepsinostreptin from *Streptomyces* sp. ACT232, isolated from deep-sea sediment collected at a depth of 1174 m in the Sagami Bay, Japan [79]. All the compounds tested in this study displayed moderate inhibitory activity against cathepsin B, with IC_50_ values ranging from 10 to 29 *μ*M. Cathepsin B had been reported to be a promising target for anticancer agents [90]. It was also identified that ahpatinin Ac and ahpatinin Pr had structural similarity with pepstatin, which is a potent aspartic protease inhibitor. By structural similarity, ahpatinin Ac, ahpatinin Pr, pepstatin Ac, and pepstatin Pr inhibited pepsin with IC_50_ values between 11 and 50 nM [79].

Desotamides B–D (40-42) are new antibiotics belonging to the cyclohexapeptides class and together with a known desotamide obtained from a deep-sea-derived *Streptomyces scopuliridis* SCSIO ZJ46, recovered from sediment sample collected at a depth of 3536 m in the South China Sea [80]. The investigators reported that desotamide and desotamide B had shown similar antimicrobial activities against *Staphylococcus aureus* ATCC 29213, *Streptococcus pneumoniae* NCTC 7466, and MRSE shhs-E1 with MIC values of 16.0, 12.5, and 32.0 *μ*g/mL, respectively. On the other hand, all the tested compounds failed to display cytotoxicities (IC_50_ > 100 *μ*M) against four human tumour cell lines SF-268, MCF-7, NCI–H460, and HepG-2 [80]. Therefore, the compounds are proposed to be promising candidatures for antibacterial drug development. The investigators suggested that the presence of Trp moiety in their defined structure is significant and might contribute to their antibacterial activity properties and made it a vital structure-activity relationship for developing new drug leads against bacterial infections.

Genome mining of *Streptomyces atratus* SCSIO ZH16 yielded a new antibiotic atratumycin (43) belonging to cyclodepsipeptide. The strain was isolated from the deep-sea sediment collected at a depth of 3536 m in the South China Sea. Atratumycin exhibited inhibitory activities against *Mycobacteria tuberculosis* H37Ra and H37Rv with MICs of 3.8 and 14.6 *μ*M, respectively [41]. The authors ascertain that atratumycin might be an excellent drug lead to be developed against tuberculosis.

### 12.3. Novel Antibacterial and Anticancer Compounds from Cultured Desert *Streptomyces*

**Figure 2:**
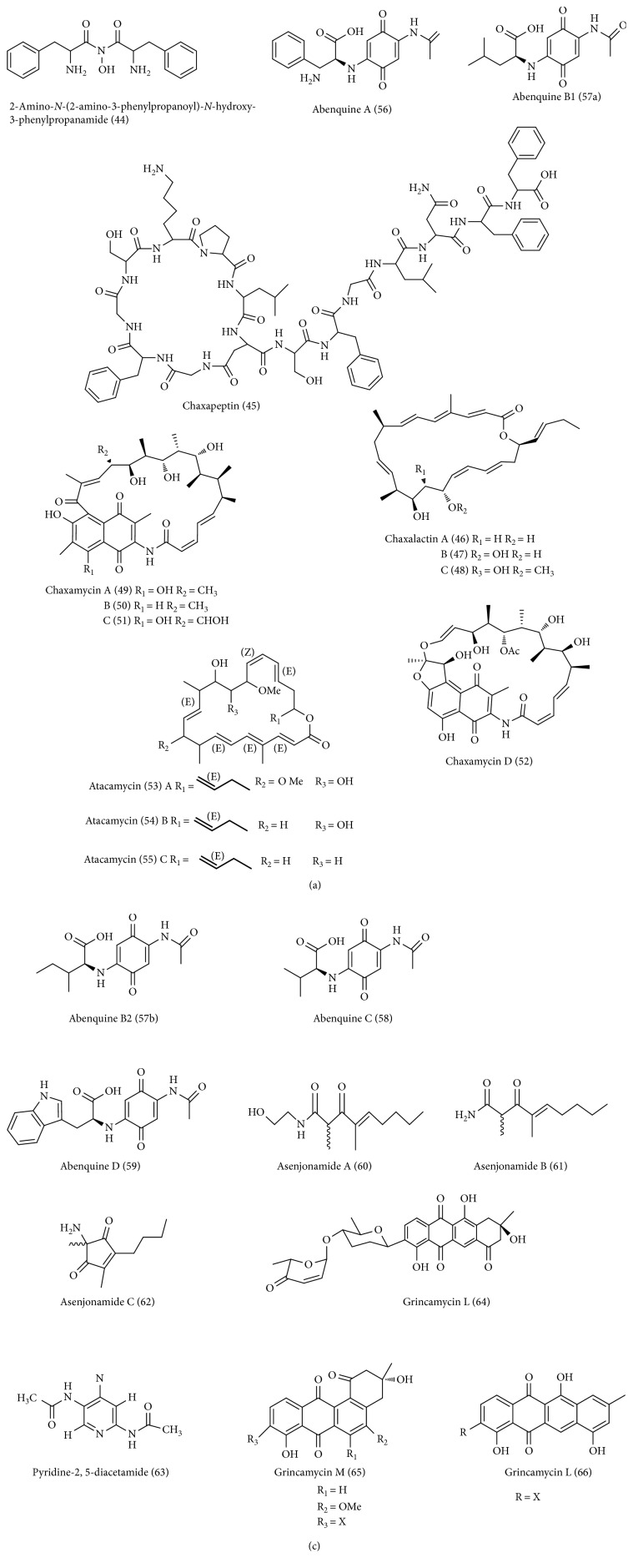
Desert *Streptomyces*-derived novel antibacterial and anticancer compounds.

Cytotoxic and antibacterial molecules derived from desert *Streptomyces* with distinct bioactivities to date are listed in Table 1, and their corresponding structures are shown in Figure 2.

2-Amino-N-(2-amino-3-phenylpropanoyl)-N-hydroxy-3-phenylpropanamide (44) is a novel hydroxamic acid-containing molecule produced by a desert *Streptomyces* strain WAB9, isolated from Saharan soil in Algeria [87]. This molecule displayed antimicrobial activity against a selection of drug-resistant bacteria, filamentous fungi, and yeasts with appreciable MICs [87].

Chaxapeptin (45) is a new lasso peptide antibiotic isolated from the fermentation broth of *Streptomyces leeuwenhoekii* strain C58, recovered from the Atacama Desert [88]. Chaxapeptin showed inhibitory activity in a cell invasion assay with human lung cancer cell line A549. Besides, this molecule has also shown weak antibacterial activity against Gram-positive bacteria, *Staphylococcus aureus*, and *Bacillus subtilis* with the MIC values of 30−35 *μ*g mL^−1^ [88].

A desert-derived *Streptomyces* sp. strain C34, isolated from a soil sample collected in Chilean hyper-arid Atacama Desert, produced three new 22-membered macrolactone polyketides, named chaxalactins A–C (46–48), together with three known compounds, deferoxamine E, hygromycin A, and 5”-dihydrohygromycin A [16]. Chaxalactins A–C exhibited strong antibacterial activity against Gram-positive bacteria with MIC values from <1 *μ*g mL^−1^against *S. aureus* and 3–6 *μ*g mL^−1^ against *L. monocytogenes*, and *B. subtilis*. But these compounds showed weak activity against Gram-negative strains tested [16].

Chaxamycins A–D (49–52) is a new ansamycin-type polyketides antibiotics isolated from the fermentation broth of *Streptomyces* sp. strain C34, recovered from a soil sample collected in the Atacama Desert [23]. Among the compounds tested, chaxamycin D showed promising selective antibacterial activity against *S. aureus* ATCC 25923 and a panel of MRSA clinical isolates.

*Streptomyces* sp. C38, isolated from the Atacama Desert, provided three new 22-membered macrolactone antibiotics named atacamycins A–C (53–55). All these compounds tested showed moderate inhibitory activity against phosphodiesterase (PDE-4B2), while atacamycin A exhibited moderate activity against adenocarcinoma and breast tumour cell lines [86].

Abenquines A–D (56-59) is a new aminoquinone-type antibiotics isolated from the fermentation broth of *Streptomyces* sp. strain DB634, recovered from a soil sample collected in the Atacama Desert [59]. All of the compounds tested displayed moderate antibacterial activity against *Bacillus subtilis*, dermatophytic fungi. Further, abenquines A and D showed moderate enzyme inhibitory activity against phosphodiesterase type 4b (PDE4b).

Asenjonamides A–C (60–62) is a new polyketide antibiotic isolated from the fermentation broth of *Streptomyces asenjonii* KNN 42.f, recovered from a soil sample collected in the hyper-arid Atacama Desert [21]. Asenjonamides A–C displayed significant antibacterial activity against Gram-positive strains of *S. aureus*, *B. subtilis*, and *E. faecalis*. Remarkably, asenjonamides C showed potent activity against Gram-negative *E. coli* to tetracycline (positive control).

*Streptomyces* sp. DA3-7, isolated from the Saudi Arabian Desert, provided a new pyridine alkaloid antibiotic named pyridine-2,5-diacetamide (63). The compound showed antibacterial activity against *Escherichia coli* and *Cryptococcus neoformans* with the MIC value of 31.25 *μ*g/mL [63].

Grincamycins L–N (64–66) is a new angucycline-type C-glycoside antibiotic isolated from the fermentation broth of *Streptomyces* sp. XZHG99 T, recovered from a soil sample collected in Color Desert, Dengpa District, Tibet [89]. Grincamycins L–N displayed significant cytotoxicity against a panel of human cancer cell lines A549, H157, MCF7, MDA-MB-231, and HepG2 [89].

### 12.4. Novel Antibacterial and Anticancer Compounds from Cultured Low Cold Environment *Streptomyces*

**Figure 3:**
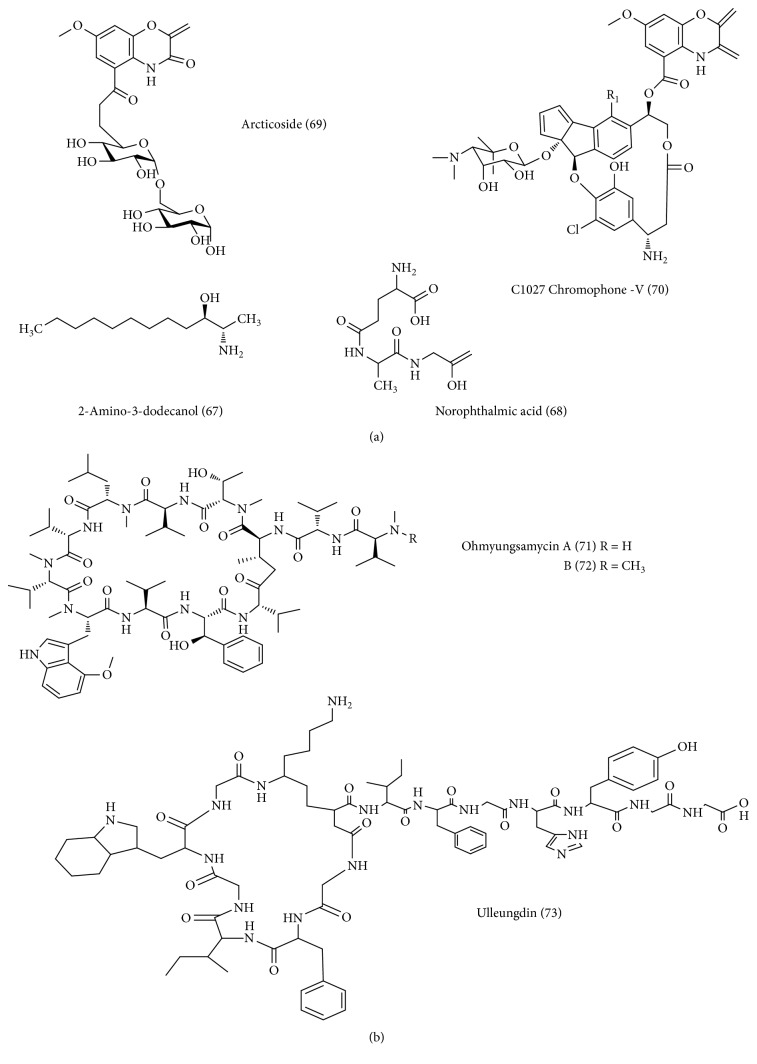
(a) Low cold *Streptomyces*-derived novel antibacterial and anticancer compounds. (b) Volcanic *Streptomyces*-derived novel antibacterial and anticancer compounds.

Table 1 and Figure 3(a) show the new bioactive molecules isolated from cryoenvironment-derived *Streptomyces*.

*Streptomyces avidinii* strain SB9 isolated from permafrost soil samples collected in Spitsbergen, Arctic Ocean, yielded three compounds named 2-amino-3-dodecanol (67) and norophthalmic acid (68) [30]. The tested compounds displayed inhibitory activity against Gram-positive bacteria and fungi.

*Streptomyces* sp. ART5, isolated from a sediment sample collected in the East Siberian continental margin of Arctic Ocean, yielded two benzoxazine antibiotics named arcticoside (69) and C-1027 chromophore-V (70) together with C-1027 chromophore-III and fijiolides A and B [69]. Arcticoside and C-1027 chromophore-V showed inhibitory activity against *Candida albicans* isocitrate lyase. But C-1027 chromophore-V exhibited significant cytotoxicity against breast carcinoma MDA-MB231 cells and colorectal carcinoma cells (line HCT-116), with the IC_50_ values of 0.9 and 2.7 *μ*M, respectively [69].

### 12.5. Novel Antibacterial and Anticancer Compounds from Cultured Volcanic Environment *Streptomyces*

Table 1 and Figure 3(b) present the new bioactive molecules isolated from cryoenvironment-derived *Streptomyces*.

Ohmyungsamycins A (71) and B (72) are new cyclic peptides isolated from the fermentation broth of *Streptomyces* sp. SNJ042, recovered from Jeju, a volcanic island in the Republic of Korea [35]. Ohmyungsamycin A showed potent cytotoxicity against various cancer cell lines such as HCT-116, A549, SNU-638, MDA-MB-231, and SKHEP-1 cells, with IC_50_ values between 359 and 816 nM. But ohmyungsamycin B exhibited weak cytotoxicity against the tested cancer cells, with IC_50_ values ranging from 12.4 to 16.8 *μ*M. Besides, ohmyungsamycin A exhibited significant inhibitory activity against selected Gram-positive and Gram-negative bacteria [35]. However, ohmyungsamycin B displayed weak antibacterial activity than ohmyungsamycin A. Further, to prove the structure and functional activity, the authors proposed that the presence of additional N-methyl group at the terminus of ohmyungsamycin B could be the possible reason for decreased bioactivity.

Ulleungdin (73) is a new 15-mer class II lasso peptide with a threaded structure isolated from the fermentation broth of *Streptomyces* sp. KCB13F003 recovered from Ulleung Island (a small volcanic island), Korea [37]. Ulleungdin exhibited significant inhibitory activities against cancer cell invasion and migration of human lung carcinoma A549 cells. The authors ascertain that ulleungdin has a low similarity (33.3%) with chaxapeptin and sungsanpin which were reported to have cancer cell invasion and migration activities. Moreover, the length of the amino acid or the size of the macrolactam ring in ulleungdin might be attributed to the anti-invasion activities [37].

## 13. Biosynthetic Gene Clusters

Biosynthetic gene cluster (BGC) containing a group of genes is responsible for the production of many of the bioactive metabolites in actinomycetes. It has been reported that gene clusters are likely to encode natural product biosynthetic pathways in sequenced microbial genomes [91]. In general, the size of the biosynthetic gene clusters in *Streptomyces* chromosome ranges from a few kb to 100 kb [92, 93]. It has been demonstrated that nonribosomal peptide synthetases (NRPS) and polyketide synthase (PKS) are known to be involved in the synthesis of many of the bioactive metabolites in actinomycetes [94]. Many gene clusters till date have been identified in *Streptomyces* spp., either of polyketide synthases (PKS), nonribosomal peptide synthetases (NRPS), or the hybrid PKS-NRPS.

## 14. Polyketide Synthases

Type I PKS gene cluster consists of multifunctional enzyme modules and at least three domains corresponding to a ketosynthase (KS), an acyltransferase (AT), and an acyl carrier protein (ACP) which attribute for the selection and condensation (Claisen type) of the correct extender unit of polyketide chain [94]. Besides, type I gene cluster contains genes such as ketoreductase (KR), dehydratase (DH), and enoyl reductase (EH) for specialised functions [95]. Type I gene cluster has been classified into two subclasses such as modular type I PKS and iterative type I PKS. In iterative type I PKS gene cluster, a single module attributes for all functions that are governing the polyketide chain elongation, whereas in modular type I gene cluster, one extension cycle is regulated by one particular PKS module. Type II PKS gene cluster contains a minimal PKS that comprises of three enzymes such as two keto acyl synthase subunits (KS*α* and KS*β*) and an acyl carrier protein (ACP). These enzymes have been reported to putatively control the choice of the starter unit and the number of extenders used in the synthesis of nascent polyketide chain [96].

## 15. Nonribosomal Peptide Synthetases

It has been documented that nonribosomal peptide synthetases (NRPSs) are mega enzymes usually with a multimodular structure, which catalyse the nonribosomal assembly of peptides from proteinogenic and nonproteinogenic amino acids [97, 98]. Schwarzer and Marahiel reported that an NRPS module usually contains an adenylation domain (A-domain), a peptidyl carrier protein domain (PCP-domain), and a condensation domain (C-domain) [99]. A-domain was determined to select the cognate amino acid (AA) from the pool of available substrates and generates the corresponding aminoacyl adenylate using ATP [100]. PCP-domain involves in the thioesterification of the activated amino acid. C-domain performs transpeptidation between the upstream and downstream peptidyl and aminoacyl thioesters to elongate the growing peptide chain. Also, it was found that a chain-terminating thioesterase domain (TE-domain) that is responsible for the detachment of the mature polypeptide [101]. There is involvement of several hundred substrates for protein synthesis by NRPSs in contrast to 20 amino acids which is confined to normal protein synthesis [97]. Interestingly, the biological functions of NRPS via synthesised compounds associated with the chemical nature of peptide which is correlated with the gene sequence [98].

## 16. Hybrid PKS-NRPS

The combination of PKS and NRPS modules may be present as a hybrid PKS-NRPS gene cluster [102].

## 17. Characterised Gene Clusters from Extreme *Streptomyces*

Though advances in genome sequencing, to date, very few gene clusters have been isolated and characterised by extreme environment *Streptomyces* and they are described below. Notably, several studies have focused on gene clusters from deep-sea *Streptomyces* and exploited for their biosynthetic pathways.

The type I PKS gene cluster governing synthesis of lobophorin from deep-sea *Streptomyces* sp. 12A35 was first isolated and exploited [103]. During the 2015s, a significant number of gene clusters from deep-sea *Streptomyces* sparked interest. The NRPS type gene cluster for marfomycin biosynthesis has been identified from *Streptomyces drozdowiczii* SCSIO 10141 [104]. Another study has demonstrated that the identification of NRPS type gene cluster responsible for the biosynthesis of desotamides by a deep-sea *Streptomyces scopuliridis* SCSIO ZJ46 [105]. The cryptic gene cluster is about 25 kb in size that is responsible for the biosynthesis of fredericamycin A (FDM A) from the mutant strain genome of *Streptomyces somaliensis* SCSIO ZH66 RIF1 which was identified by Zhang et al. [106]. The type I PKS heronamide gene cluster from deep-sea *Streptomyces* sp. SCSIO 03032 was isolated and characterised [107]. Recently, Ma and coworkers identified spiroindimicin (SPM) gene cluster from *Streptomyces* sp. SCSIO 03032 [108]. A recent study explored the atratumycin biosynthetic gene cluster from *Streptomyces atratus* SCSIO ZH16 [41]. The gene clusters for chaxamycin, chaxalactin, and chaxapeptin biosynthesis have been identified from *S. leeuwenhoekii* C34 recovered from the Atacama Desert [109]. A recent study demonstrated the gene cluster responsible for ulleungdin from *Streptomyces* sp. KCB13F003 isolated from Ulleung volcanic Island [37].

## 18. Conclusion and Future Remarks

In conclusion, to date reports suggest that extreme *Streptomyces*-derived natural compounds with their structure-activity relationship (SAR) have an incredible source to develop future drugs against cancer and bacterial infections. Thus, it becomes clear that potential *Streptomyces* are existed in all the extreme environments so far studied. Furthermore, the future identification of various gene clusters from extreme habitat-derived *Streptomyces* unlocks the different hidden natural products biosynthetic machinery in more detail and would make it possible for combinatorial biosynthesis to expand more natural products with distinct structural diversity. Furthermore, the whole-genome sequence (WGS) analysis of the potent strains would provide an insight into how these strains adapt to extreme environmental conditions and different regulatory pathways that are associated with bioactive compound productions.

Though there is evidence that interest sharply decreased in natural product discovery in the past decades, the future would largely depend on academic and biotech industries collaboration. The present review also highlighted that research on extreme habitat *Streptomyces*-derived natural products constantly continued to grow in the specific geographical location especially in the South China Sea, Atacama Desert, Arctic, and Korean volcanic regions. It is the hope that additional report will become available from other extreme areas over time in respect of novel natural compounds. Therefore, the authors ascertained herein that *Streptomyces* from extreme habitat will be an excellent source of novel antibiotics with distinct biological activities in the fight against bacterial infections and cancer.

## Figures and Tables

**Table 1:** Antibacterial and anticancer compounds derived from deep-sea, desert, low cold, and volcanic *Streptomyces*.

Deep-sea *Streptomyces*-derived novel drugs						
Compound	Structural class	Source	Activity	Depth	Region	Reference
Caboxamycin	Benzoxazole	*Streptomyces* sp. NTK 937	Antibacterial, antifungal, antitumor	3814 m	Atlantic	[71]
Ammosamides	Pyrroloiminoquinone	*Streptomyces* sp. CNR-698	Cytotoxic	1618 m	Bahamas	[72]
Spiroindimicins	Bisindole alkaloid	*Streptomyces* sp. SCSIO 03032	Cytotoxic	3412 m	Indian Ocean	[73]
Indimicins	Bisindole alkaloid	*Streptomyces* sp. SCSIO 03032	Cytotoxic	3412 m	Indian Ocean	[74]
Grincamycins	Glycoside	*Streptomyces lusitanus* SCSIO LR32	Cytotoxic	3370 m	South China Sea	[75]
Lobophorins E and F	Spirotetronate	*Streptomyces* sp. SCSIO 01127	Cytotoxic, antibacterial	1350 m	South China Sea	[76]
Lobophorins H and I	Spirotetronate	*Streptomyces* sp. 12A35	Antibacterial	2134 m	South China Sea	[46]
Marfuraquinocins	Sesquiterpenoid naphthoquinones	*Streptomyces niveus* SCSIO 3406	Cytotoxic, antibacterial	3536 m	South China Sea	[77]
Sungsanpin	Peptide	*Streptomyces* sp. SNJ013	Inhibitory activity to A549 with the cell invasion assay	138 m	Jeju Island	[78]
Ahpatinin	Peptide	*Streptomyces* sp. ACT232	Aspartic protease inhibitors	1174 m	Sagami Bay	[79]
Desotamides B−D	Peptides	*Streptomyces scopuliridis* SCSIO ZJ46	Antibacterial	3536 m	South China Sea	[80]
Marangucyclines A and B	Angucycline	*Streptomyces* sp. SCSIO 11594	Cytotoxic, antibacterial	2403 m	South China Sea	[47]
Grincamycin H	Glycoside	*Streptomyces lusitanus* SCSIO LR32	Cytotoxic	3370 m	South China Sea	[81]
Fradiamines A and B	Siderophore	*Streptomyces fradiae* MM456M-mF7	Antibacterial	806 m	Sagami Bay	[82]
Lobophorin K	Lobophorin	*Streptomyces* sp. M-207	Cytotoxic	1800 m	Central Cantabrian Sea.	[83]
Spiroindimicins G and H	Bisindole alkaloid	*Streptomyces* sp. SCSIO 03032	Anticancer	3412 m	South China Sea	[84]
Atratumycin	Cyclodepsipeptide	*Streptomyces atratus* SCSIO ZH16	Antituberculosis	3536 m	South China Sea	[41]
3-Hydroxyquinaldic acid derivative		*Streptomyces cyaneofuscatus* M-157	Cytotoxic	2000 m	Central Cantabrian Sea	[85]
Anthracimycin B	Macrolide	*Streptomyces cyaneofuscatus* M-169	Antibacterial	1500 m	Cantabrian Sea	[48]

Desert *Streptomyces*-derived novel antibacterial and cytotoxic drugs						
Compound	Structural class	Source	Activity	Region	Reference	

Chaxalactins	Macrolactone polyketides	*Streptomyces* sp. strain C34	Antibacterial	Atacama	[16]	
Chaxamycins	Ansamycin-type polyketides	*Streptomyces* sp. strain C34	Antibacterial	Atacama	[23]	
Atacamycins	Macrolactone	*Streptomyces* sp. C38	Enzyme inhibitor, antiproliferative	Atacama	[86]	
Abenquines	Aminoquinone	*Streptomyces* sp. strain DB634	Enzyme inhibitor for phosphodiesterase type 4b	Atacama	[59]	
2-Amino-*N*-(2-amino-3-phenylpropanoyl)-*N*-hydroxy-3-phenylpropanamide	Hydroxamic acid	*Streptomyces* strain WAB9	Antimicrobial	Saharan	[87]	
Chaxapeptin	Peptide	*Streptomyces leeuwenhoekii* strain C58	Inhibitory activity in a cell invasion assay with A549	Atacama	[88]	
Asenjonamides	Polyketide	*Streptomyces asenjonii* KNN 42.f	Antibacterial	Atacama	[21]	
Pyridine-2,5-diacetamide	Pyridine alkaloid	*Streptomyces* sp. DA3-7	Antibacterial	Saudi Arabian Desert	[63]	
Grincamycins	Angucycline	*Streptomyces* sp. XZHG99 T	Cytotoxic	Color Desert	[89]	

Low cold *Streptomyces*-derived antibacterial and cytotoxic drugs						
Compound	Structural class	Source	Activity	Region	Reference	

2-Amino-3-dodecanol, norophthalmic acid, phthalic acid ester		*Streptomyces avidinii* SB9	Antibacterial	Arctic	[30]	
Arcticoside, C-1027 chromophore-V	Benzoxazine	*Streptomyces* sp. ART5	Cytotoxicity activity	Arctic	[69]	

Volcanic *Streptomyces*-derived antibacterial and cytotoxic drugs						
Compound	Structure	Source	Activity	Region	Reference	

Ohmyungsamycins A and B	Peptide	*Streptomyces* sp. SNJ042	Antibacterial, cytotoxic	Korean volcanic	[35]	
Ulleungdin	Lasso peptide	*Streptomyces* sp. KCB13F003	Inhibited the invasion and migration of human lung carcinoma A549	Korean volcanic	[37]	
